# Arts-based approaches to promoting health in sub-Saharan Africa: a scoping review

**DOI:** 10.1136/bmjgh-2019-001987

**Published:** 2020-05-21

**Authors:** Christopher Bunn, Chisomo Kalinga, Otiyela Mtema, Sharifa Abdulla, Angel Dillip, John Lwanda, Sally M Mtenga, Jo Sharp, Zoë Strachan, Cindy M Gray, Culture and Bodies’ Team, Amelia Crampin

**Affiliations:** 1 College of Social Sciences, University of Glasgow, Glasgow, UK; 2 Social Sciences, Malawi Epidemiology and Intervention Research Unit, Lilongwe, Malawi; 3 Centre of African Studies, University of Edinburgh, Edinburgh, UK; 4 Zaluso Arts, Lilongwe, Malawi; 5 Department of Fine and Performing Arts, Chancellor College, Zomba, Malawi; 6 Health Systems, Impact Evaluation and Policy, Ifakara Health Institute, Dar es Salaam, United Republic of Tanzia; 7 School of Geography and Sustainable Development, University of St Andrews, St Andrews, UK; 8 School of Critical Studies, University of Glasgow, Glasgow, UK

**Keywords:** health education and promotion, health policy, review

## Abstract

**Introduction:**

Arts-based approaches to health promotion have been used widely across sub-Saharan Africa (SSA), particularly in public health responses to HIV/AIDS. Such approaches draw on deep-rooted historical traditions of indigenous groups in combination with imported traditions which emerged from colonial engagement. To date, no review has sought to map the locations, health issues, art forms and methods documented by researchers using arts-based approaches in SSA.

**Methods:**

Using scoping review methodology, 11 databases spanning biomedicine, arts and humanities and social sciences were searched. Researchers screened search results for papers using predefined criteria. Papers included in the review were read and summarised using a standardised proforma. Descriptive statistics were produced to characterise the location of the studies, art forms used or discussed, and the health issues addressed, and to determine how best to summarise the literature identified.

**Results:**

Searches identified a total of 59 794 records, which reduced to 119 after screening. We identified literature representing 30 (62.5%) of the 48 countries in the SSA region. The papers covered 16 health issues. The majority (84.9%) focused on HIV/AIDS-related work, with Ebola (5.0%) and malaria (3.3%) also receiving attention. Most studies used a single art form (79.0%), but a significant number deployed multiple forms (21.0%). Theatre-based approaches were most common (43.7%), followed by music and song (22.6%), visual arts (other) (9.2%), storytelling (7.6%) and film (5.0%).

**Conclusions:**

Arts-based approaches have been widely deployed in health promotion in SSA, particularly in response to HIV/AIDS. Historically and as evidenced by this review, arts-based approaches have provided a platform to facilitate enquiry, achieved significant reach and in some instances supported demonstrable health-related change. Challenges relating to content, power relations and evaluation have been reported. Future research should focus on broadening application to other conditions, such as non-communicable diseases, and on addressing challenges raised in research to date.

**Key questions**

**What is already known?**

- Arts-based approaches to health improvement have been used across sub-Saharan Africa (SSA), with some studies reporting positive outcomes.
- No comprehensive scoping review of this work has been produced.
- A rich array of approaches to using arts in support of health have been pursued in the global north, but the decolonising methodology movement suggests we should be careful not to import these into global south contexts.

**What are the new findings?**

- Our review suggests that an overwhelming majority of studies have focused on HIV/AIDS, and that theatre-based approaches were the most common, but music and song, visual arts, storytelling and film have received sustained attention in the research literature.
- Arts-based approaches have facilitated research enquiry, reach large numbers with health-promoting messages and initiatives, and in some instances supported demonstrable health-related change.
- Challenges relating to the generation and suitability of content, power relations between researchers and target community and appropriate forms of evaluation have been reported.

**What do the new findings imply?**

- In SSA, arts-based approaches to health promotion have yet to be widely applied by researchers outside of HIV/AIDS, suggesting that future research could develop approaches to non-communicable diseases, neglected tropical diseases and communicable diseases other than HIV/AIDS.
- There is great scope to develop theories of and methodologies for arts-based approaches to health in SSA that dialogue with, but do not mimic, those developed in the global north.

## Introduction

A long-standing critique of health promotion and intervention is that many practitioners uncritically impose categories and understandings from biomedicine on the communities with which they work.^1 2^ Yet, efforts to facilitate improved health in a community are inevitably faced with the ideas, priorities and concerns that members of that community attach to health.^3^ Failure to acknowledge, respect and work with the situated cultural positions held by community members can lead to cultural imposition,^4^ introjection^5^ and symbolic violence,^6^ which undermine the pursuit of better health. A range of researchers and practitioners have sought to address these problems by developing approaches to health promotion^7^ inspired by action research^8^ and dialogical theory.^9^ Such approaches treat health promotion and intervention as a collaboration between community members, healthcare professionals and researchers.^10^

One way in which researchers have pursued collaboration with communities to improve health in sub-Saharan African (SSA) is by using artistic forms.^11–13^ In SSA, creative processes have been harnessed to *enquire* into local experiences and understandings relating to health,^14^ to *intervene* in drivers of health problems,^15^ to provide a discreet form of *therapy* for a range of conditions^16^ and to *disseminate and validate* health research findings.^17^ These four modes have indigenous roots in African communities.^18^ As Kerr notes, indigenous traditions such as the *Gule wa Mkulu* have long served as a space for moral education (eg, the dangers of excessive alcohol consumption) and the negotiation of social change in Chewa and Manganja communities.^19^ Members of this SSA research community make the claim that artistic forms and methods facilitate participants to step outside of their everyday lives to use imagination and play to reconfigure how they understand and act on an aspect of life, such as a disease.^20^ They also point to the importance of the potential for art to engage communities in embodied learning,^21^ to stimulate empathy^22^ and to provide vicarious experiences that shape collective and self-perceptions.^23^

Building on indigenous performative traditions, arts-based methods have featured prominently in public health action across SSA. Kamlongera points to three phases in this action: during the precolonial era, artistic expression, or ‘folk media’, was central to cultural transmission and education in many communities across Africa; during the colonial era, these folk media were often appropriated for the purposes of ‘moral education’, notably through the ‘propaganda play’ and in the postcolonial era, there has been a resurgence of interest in art and folk media as media for education and development.^12^ Of the art forms, theatre has been widely used by governments in SSA in the postcolonial era, as it is seen as a ‘convenient instrument’ through which to influence health-related practices in contexts of low levels of literacy, receiving significant funding.^24^

Using the arts for health in the postcolonial era has been most visible in responses to the HIV and AIDS epidemic. Practitioners of theatre, song, storytelling, dancing, circus and many other artistic forms have made substantial contributions to awareness raising, prevention, testing, destigmatising and counselling initiatives.^13^ In recent years, the Ebola epidemic has also received attention from the artistic community, notably in West Africa, which sought to communicate the danger posed by the virus and how to respond to symptoms.^25^ While the number of studies taking such approaches to HIV and AIDS and other health issues is large, no attempt has been made to systematically map the locations, health issues, art forms and methods relating to this body of work. Our review seeks to redress this gap in the literature and to provide researchers, practitioners and policy makers with an overview of the achievements and challenges identified in studies to date.

In doing so, we situate ourselves within the ‘decolonising methodologies’ movement^26^ and attempt to avoid imposing frameworks from the rich array of arts-based research conducted in the global north.^27^ For this reason, our review is focused on the use of art in the pursuit of the World Health Organisation’s (WHO) widely accepted definition of health promotion, from the Ottawa Charter of 1986: ‘health promotion is the process of enabling people to increase control over, and to improve, their health.’^28^ Our application of the WHO approach led us to exclude arts-based approaches to research (except when part of a health promotion effort),^29^ therapy^30^ and knowledge translation.^31^ For, while these three traditions often overlap and intersect with health promotion, their primary aims are discreet.

## Methods

We adopted scoping review methodology, as defined by Arksey and O’Malley^32^ and Levac *et al*,^33^ to enable an iterative and exploratory approach to knowledge gathering and synthesis. Specifically, we followed the five stages proposed by Arksey and O’Malley:

1. Identifying the research question.
2. Identifying relevant studies.
3. Study selection.
4. Charting the data.
5. Collating, summarising and reporting results.

Unlike systematic reviews, scoping reviews do not seek to answer narrow questions with the best available evidence, but instead map what is known about a topic, identify gaps in research literature and inform future investigations.^34^

### Identifying the research question

Although health promotion in SSA has often used artistic forms, an encompassing review of the academic literature that these initiatives have generated has never, to our knowledge, been undertaken. To address this gap, we sought to answer the broad research question: how have arts-based approaches been used to promote health in SSA? Beneath this question, we also set out to explore the achievements and challenges that the existing literature reports on, and to produce a synthesis of learning points from the corpus as a whole.

### Identifying relevant studies

Arts-based health promotion frequently involves collaboration between researchers and practitioners from the arts, social sciences and biomedicine. For this reason, we searched a wide variety of databases representing the plurality of disciplines. Preliminary searches were conducted using the EBSCOhost and ProQuest platforms and the search strategy was refined iteratively to ensure that a selection of eight key papers, identified through discussion among the authors, were returned by the strategy. The searches were limited to papers published in English and included search terms (see online supplementary file 1, table 1) that limited geography to SSA territories (using the World Bank’s definition of the SSA region),^35^ applying both current and historical names for territories in the region. The final search terms were established in consultation with a librarian before two researchers (CB and OM) searched 11 databases (see online supplementary file 1, table 2). Searches were carried out in May 2018, with no limitations placed on date of publication. Finally, the bibliographies of all papers that were included in the review were searched by hand for references not identified during the database searches.

10.1136/bmjgh-2019-001987.supp1 Supplementary data

During preliminary searches, we found that our search strategy returned papers relating to television (TV) and radio productions, triggered by the inclusion of the term ‘drama’ in the search. While there is a polarising and long-standing debate about whether TV and radio can be considered ‘art’, we settled on an inclusive strategy that included these cultural forms.^36^

### Study selection

Titles were screened for records that appeared to represent efforts to use artistic practices to promote health in SSA territories. Three researchers (CB, CG and OM) screened record titles in separate EndNote libraries. These libraries were then combined to form a second library containing the records selected for abstract screening. The following inclusion and exclusion criteria for abstract and full texts were applied:

1. Papers set in SSA.
2. Papers that report using arts.
3. Papers with a focus on health promotion/intervention.

#### Not

1. Papers from non-SSA territories.
2. Papers that use arts to recruit to clinical interventions or disseminate research findings.
3. Papers that focus on community art without relating this to health.

The database of records was divided between eight researchers (AD, CB, CMG, CK, JL, OM, SA and SM), who worked in pairs to screen the abstracts for inclusion. Two researchers (CB and OM) reviewed the decisions made by the paired researchers and created a list of records for full-text review. These records were then provided to seven researchers (AD, CB, CMG, JL, JS, OM and SA) who read the papers in full and proceeded to extract data from those papers that met the review inclusion criteria. Decisions and extractions were reviewed by two authors (CB and OM).

### Charting the data

Given the interdisciplinary nature of the enquiry and the multiple disciplines from which the records were drawn, we used a template that was broad enough to encompass approaches that did not fit the ‘headings’ under which traditional biomedical/public health enquiry is conducted. The template included the following fields:

- Author(s), year of publication, study location and study duration.
- Study community, target group and/or sample.
- Aims of the study or paper.
- Art forms used/discussed, and health issue focused on.
- Methodology/approach
- Activities/interventions.
- Analysis undertaken/outcome measures.
- Study results, key arguments, key messages.

The researchers attempted to ensure that a uniform approach to data charting was achieved, but in practice the variety of approaches taken in this cross-disciplinary literature meant that some information was not available for all records. Two authors (CB and OM) reviewed and supplemented the content captured during the charting stage.

### Collating, summarising and reporting results

The content generated during charting phase was combined in an Excel database, which was used to generate descriptive statistics relating to the location of the studies, year of publication, art forms used or discussed and the health issues addressed. Graphs and visualisations were produced at this stage, to aid interpretation of the data.

After consideration of potential approaches to summarising and reporting the data, we chose to present the data by art form, as to do so by health issue or location would have led to clumping in the HIV/AIDs category. Our initial approach to categorising art forms was grounded, following the labels applied by the authors of the papers we reviewed. During the process of summarising the data, we maintained these grounded labels, with the exception of three groups of art forms: theatre, visual arts and digital arts. These groupings aggregate a range of author-applied labels. Our reporting combines multiple groupings (indicated by the use of a comma in the subheading).

Our approach to reporting the results below draws on the narrative review tradition, which is a common and accepted approach to reporting findings of a scoping review.^37^ Adopting narrative-based reporting enabled our multidisciplinary team to participate in the analysis and presentation of the diverse set of methodological and disciplinary traditions present in the literature we identified.^38^

### Patient and public involvement

No patients or members of the public were involved in the design of this literature review.

## Results

### Scoping review search and initial screening

**Figure 1:**
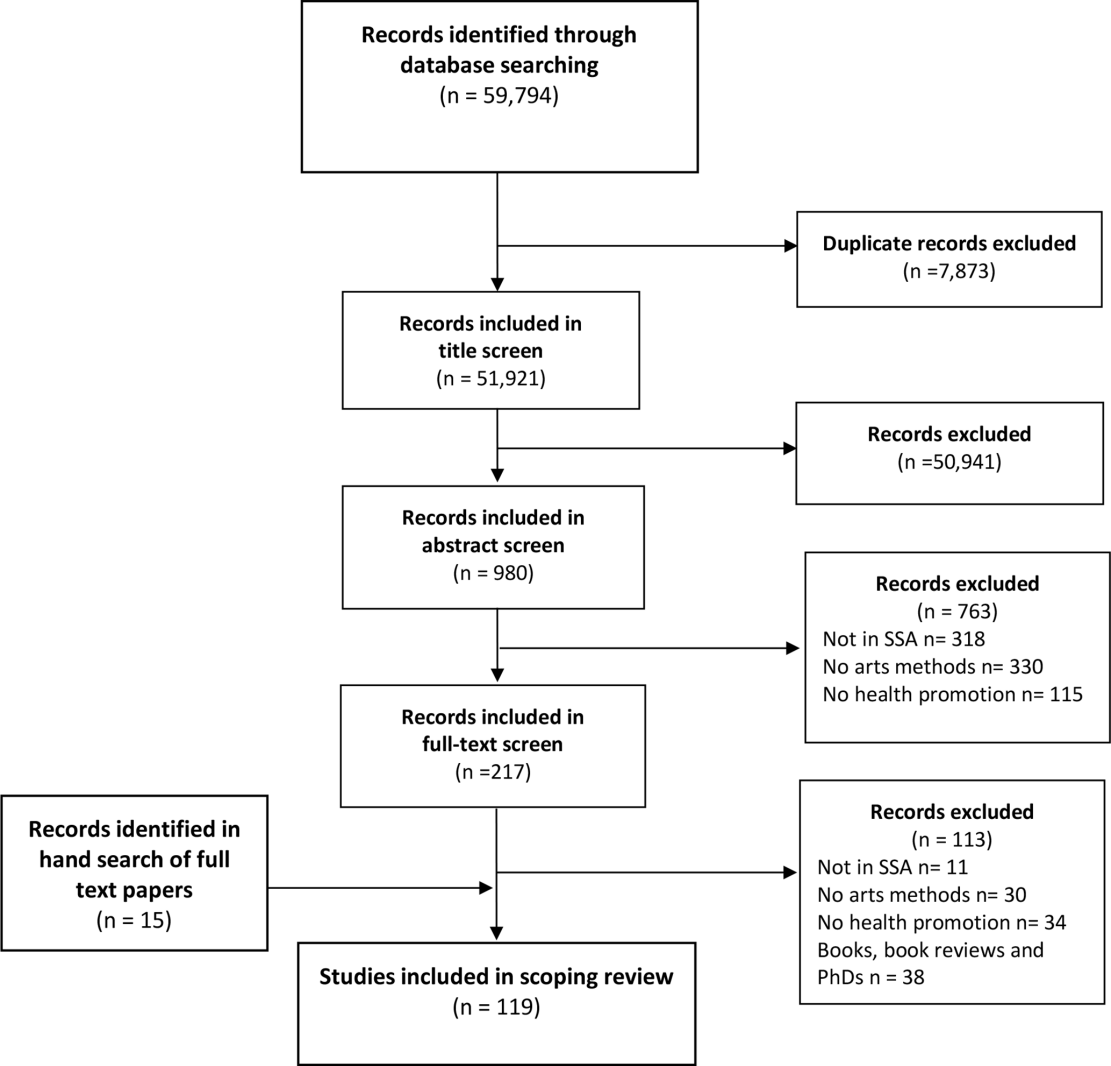
Flow diagram of review. SSA, sub-Saharan Africa.

Online supplementary table 1 details the database search, which identified 59 794 records. After de-duplication, 51 921 records remained. Screening of record titles excluded 50 941 records. The abstracts of the remaining 980 articles were screened and differences of opinion relating to 9% of records were resolved through discussion. As figure 1 shows, 763 records were excluded at the abstract stage, and 217 records included for full-text screening. During the full-text screening stage, 113 records were excluded, leaving 104 for data extraction. A further 15 papers were identified in the references lists of included papers, bringing the total number of records reviewed to 119.

### Description of included papers

**Figure 2:**
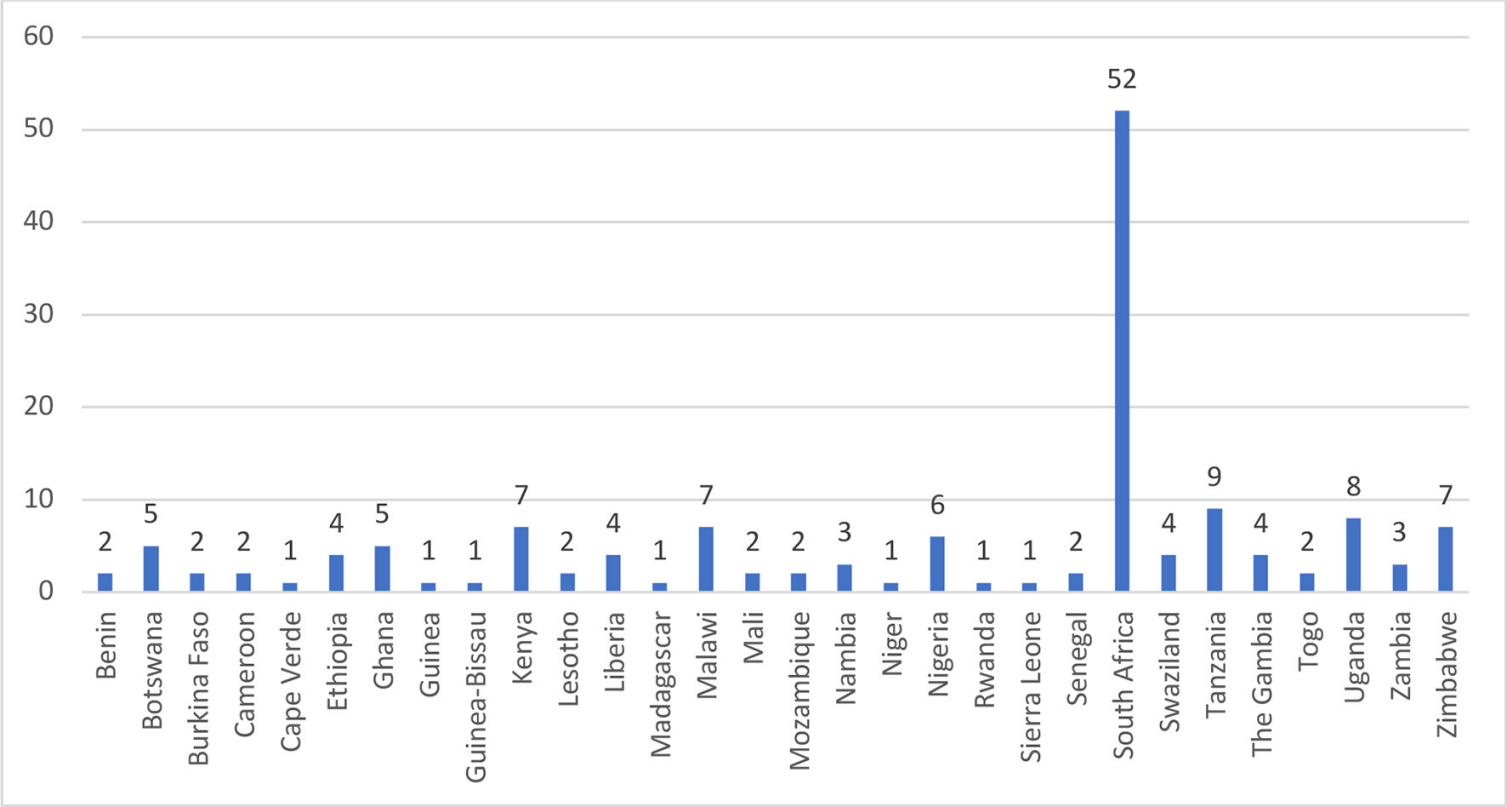
Geographical location of studies identified in the review.

Our review identified work representing 30 countries in the SSA region (see figure 2). Of the 119 studies, 52 (43.7%) related to South Africa, 9 (7.6%) to Tanzania, 8 (6.7%) to Uganda, 7 (5.9%) each to Kenya, Malawi and Zimbabwe, 6 (5.0%) to Nigeria, 5 (4.2%) to Botswana and Ghana, 4 (3.3%) each to Ethiopia, Liberia, Swaziland and the Gambia, and 3 (2.5%) to Zambia and Namibia. The remaining 15 (12.6%) countries were associated with two studies (Benin, Burkina Faso, Cameroon, Lesotho, Mali, Mozambique, Senegal and Togo) or one study (Cape Verde, Guinea, Guinea-Bissau, Madagascar, Niger, Rwanda and Sierra Leone).

**Figure 3:**
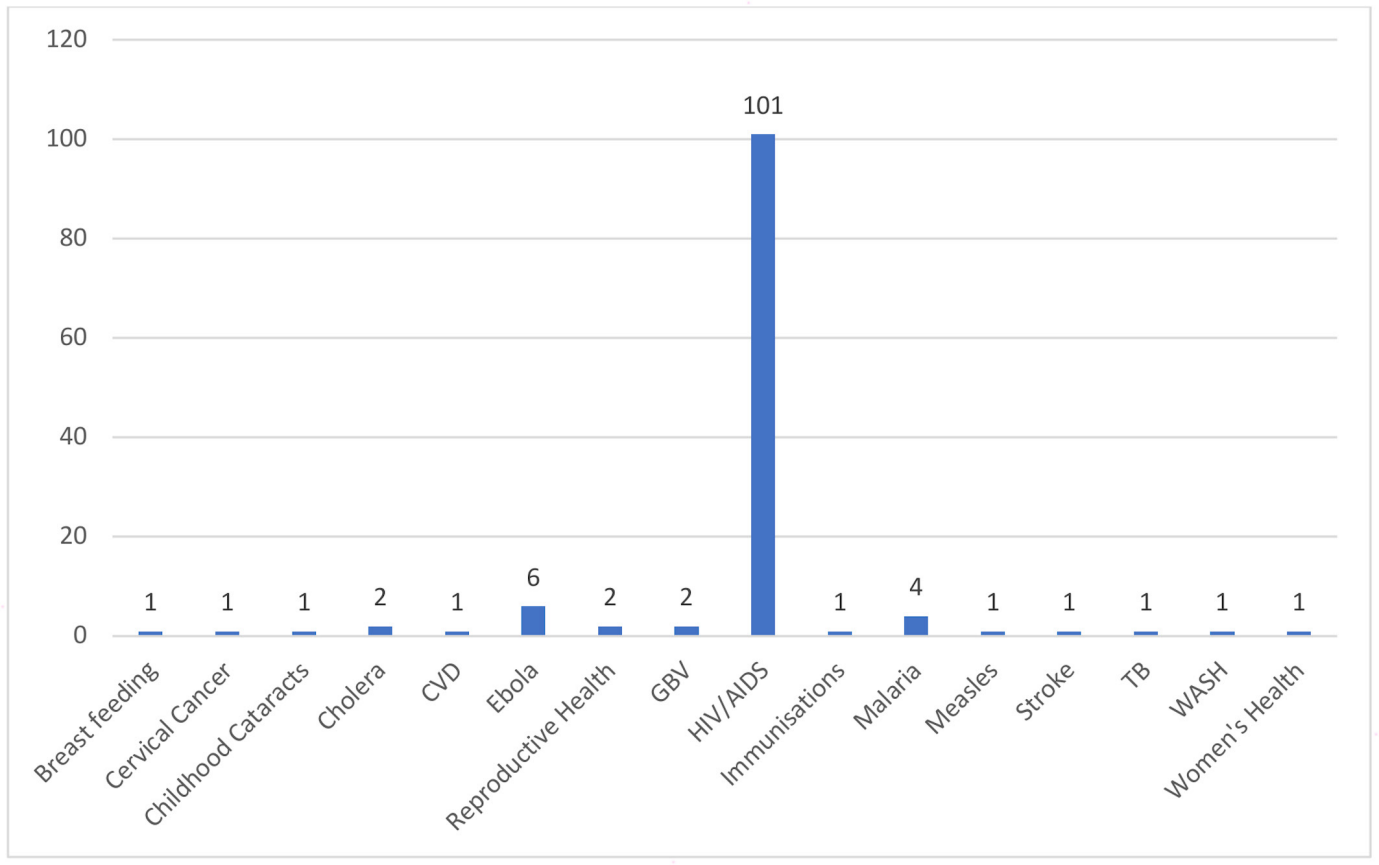
Focal health issues identified in the included records. CVD, cardiovascular disease; GBV, gender-based violence; TB, tuberculosis; WASH, water, sanitation and hygiene.

The studies addressed 16 different health issues (see figure 3). A significant majority related to HIV/AIDS (101 (84.9%)). Ebola was the subject of six (5.0%) studies, malaria four (3.3%), and cholera, reproductive health and gender-based violence two (1.7%). The remaining 10 (8.4%) health issues were covered in single papers (breast feeding, cervical cancer, childhood cataracts, cardiovascular disease, immunisation, measles, stroke, tuberculosis, water, sanitation and hygiene, and ‘women’s health’).

**Figure 4:**
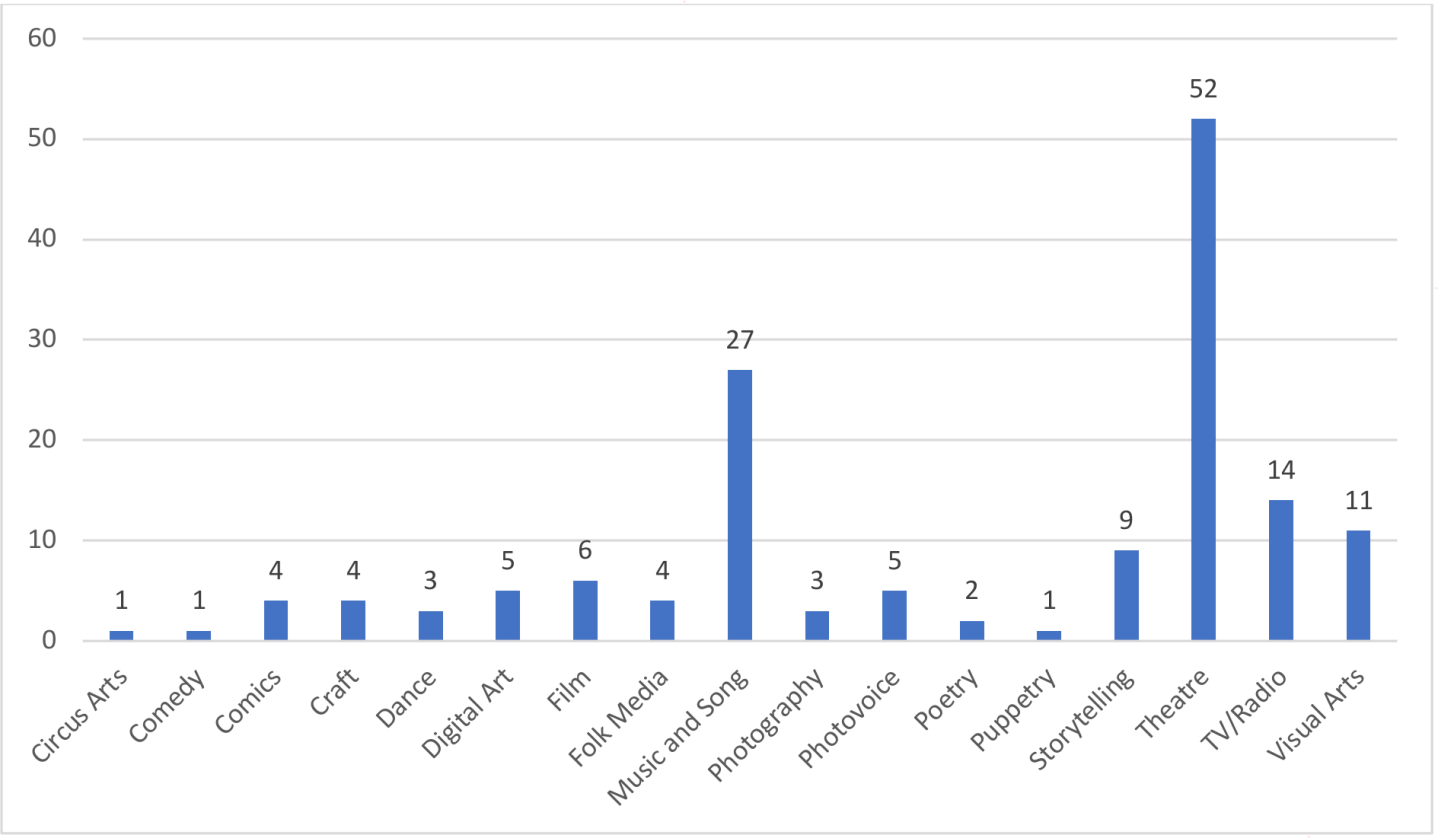
Types of art forms identified in the included records.

**Figure 5:**
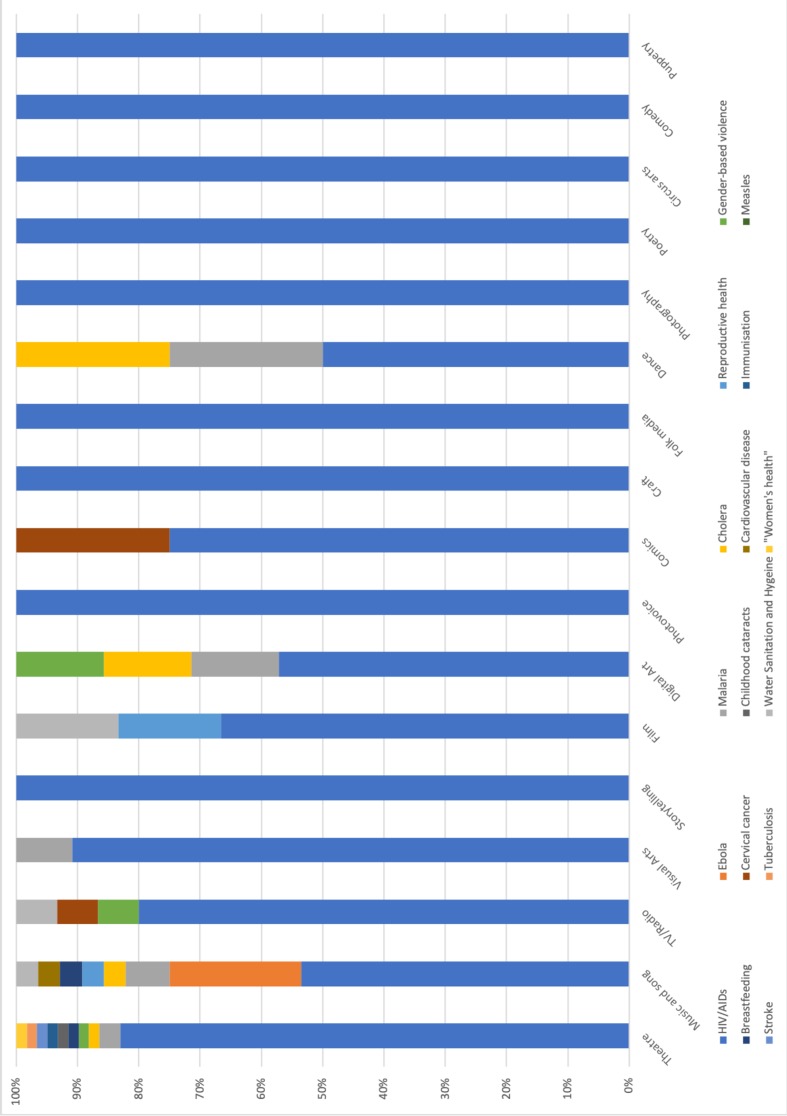
Proportional distribution of art forms by health issues.

The studies employed or addressed the use of 17 different categories of art forms (see figure 4). Theatre was present in 52 (43.7%) studies, music and song in 27 (22.6%), TV/radio in 14 (11.7%), visual arts in 11 (9.2%), storytelling in 9 (7.6%) and film in 6 (5.0%). Digital art and photovoice were referred to in five (4%); papers, comics, craft and folk media in four (3.3%); dance and photography in three (2.5%); poetry in two (1.7%); and circus arts, comedy and puppetry in one. Of the 119 studies, 25 (21.0%) used or referred to more than one artistic form. In the remainder of the paper, we refer to these studies as ‘multiform’. Figure 5 visualises the proportional distribution of art forms by health issues.

### Theatre

Among the papers identified in the review, the most common art form used to promote health was theatre. Of the 52 papers we identified,^15 17 20 39–87^ 14 (26.9%) presented theatre as part of a multiform approach. Beneath the category of ‘theatre’, many studies located themselves with reference to specific traditions or techniques: theatre for development,^82^ participatory theatre,^85^ forum theatre,^58^ image theatre,^57^ community theatre^59^ and appreciative enquiry.^73^ Papers often described an extended period in which facilitators worked with community groups to develop a performance by eliciting narratives relating to experiences and perceptions of health conditions (usually HIV/AIDS), before putting on a performance for the community.^49 51 54 55 64^

A subgroup (9 (17.3%)) of the theatre-based papers presented evaluations of interventions that included statistical assessments of outcomes.^15 40 41 45 50 55 77 78 83^ A large-scale randomised community trial of a school-based drama programme in South Africa found significant improvements in HIV/AIDS-related knowledge and attitudes, as well as condom use among those who were sexually active.^45^ Another South African study found that public theatre performances produced in collaboration with community members, working within the Xhosa educative storytelling tradition of *intsomi*, were significantly associated with large increases (172%) in self-referrals to HIV/AIDS voluntary counselling and testing services.^15^

In some instances (19/52 (36.5%)), studies used theatrical techniques to help participants explicitly explore potential solutions to health-related struggles or dilemmas.^17 41 43 44 49 57 58 61 64 66 71 73 74 78 84–86^ One paper presented role-play techniques and Boalian image theatre to act out HIV/AIDS-related scenarios, such as pressured situations in which women are expected to have unprotected sex, consider strategies for managing these situations and then role-play alternative responses, with the aim of helping participants to plan ahead for such difficult situations.^57^ Another paper described using body sculpture, image theatre and role-play to help healthcare workers explore and mitigate their risk of contracting tuberculosis (TB) while at work.^85^

Critical reflections on working with theatre-based art forms were also present in the corpus. One author questions the extent to which ‘participatory’ methods referred to in some work are truly participatory, suggesting that some invoke the term without in fact providing a space for empowerment or agency.^80^ Another paper, reflecting on community theatre approaches to HIV/AIDS prevention in Tanzania, noted that audiences sometimes reacted negatively to the open depiction of sexual matters, and that post-performance discussion were often shunned, particularly by men.^59^

### Music and song, dance

We identified 27 papers that considered music, song and/or dance as mediums through which to engage in health promotion,^25 39 50 53 60 61 77 83 88–106^ 11 (40.7%) of which were part of multiform work. One body of work (7/27 (25.9%)) we identified explored how popular artists have incorporated health-promoting messages in their songs, specifically in relation to HIV/AIDS and Ebola.^25 92 94 96 102 103 105^ A study in Tanzania used interviews to explore young peoples’ engagement with popular music and emphasised the utility of metaphor to communicate the nature of HIV/AIDS, the important role songs can play in confronting stigma and for addressing myths about who is affected by the condition.^94^ An ethnographic study in Liberia documented how popular musicians, sometimes commissioned by non-governmental organisations (NGOs) such as UNICEF or WHO, responded to the Ebola outbreak in 2014–2016 with a series of songs and ‘jingles’ which communicated preventative measures, as well as basic information about the condition.^25^

Other studies (6/27 (22.2%)) worked with members of communities to produce music and song with health-improving or preventative intentions.^90 93 95 98 101 104^ In the Gambia, one paper reports how songs that encouraged bed net repair for malaria prevention were crafted by community members in response to a focus group discussion exploring malaria and prevention.^90^ The researchers leading this study facilitated the recording and circulation of these songs, accompanied by locally crafted posters, and found statistically significant improvements in bed net repair after communities were exposed to them. In South Africa, researchers explored the use of djembe drumming circles as an intervention to promote low-intensity to moderate-intensity physical activity.^98^ The study found that drumming was safe for hypertensives and was associated with a statistically significant reduction in blood pressure.

Research on HIV/AIDS activist choirs in South Africa has been conducted in Zulu-speaking communities. Through ethnographic study, two papers present how such choirs provided both support networks for people living with HIV/AIDS and a platform for activism and preventative communication.^97 100^ In the Gambia, researchers also used ethnographic methods to examine a government-funded Ebola initiative, which engaged local *kanyeleng* female fertility societies to compose and perform Ebola communication and prevention songs and dances. The studies argue that the initiative fostered trust in the messaging and provided reach that conventional methods would not have achieved.^101 104^

All three of the papers which we identified as working explicitly with dance were part of multiform approaches.^46 81 83^ Two addressed HIV/AIDS^46 81^ and one focused on cholera and malaria.^83^ Given the lack of distinction between music, song and dance in many African traditions,^107^ it is likely that the apparently small number of dance-related studies identified in this review masks the extent to which dance was present in music and song-based studies.

### TV/radio

The third largest group of papers identified in the review related to work that used TV or radio platforms for health promotion purposes. Of the 14 papers in this group,^103 108–120^ 4 (28.6%) described multiform interventions. The dominant paradigm operating in this body of literature is ‘entertainment-education’, which describes material that self-consciously seeks to embed educational messages within mass art forms.^121^ A common study design involved the airing of a TV or radio series containing HIV/AIDS-related story lines, followed by a survey among target communities to assess potential impacts on HIV/AIDS-related practices. For example, one study explored the effect of a radio drama which aimed to increase HIV/AIDS testing rates in Botswana. In a random-sample survey of 555 people across seven districts, researchers found that nearly half of respondents listened to the drama, and that listening to the drama was positively associated with lower levels of stigma toward people living with HIV/AIDS, stronger intentions to get tested and increased discussions with partners about getting tested.^115^

In South Africa, researchers conducted a nationwide evaluation of the ‘Soul City’ drama serial, which was broadcast through both TV and Radio and supported by booklets distributed through national newspapers.^111^ The drama integrated health-promoting messages with stories from daily life in South Africa, with the aim of reducing infection rates, stigma and risky behaviour in teenagers. In a random sample national survey of 1981 people, 82% reported being exposed to Soul City via TV, radio or booklet. The survey data also suggested that both the TV and radio drama were associated with significant positive shifts in perception of social norms relating to HIV/AIDS stigma. Exposure to Soul City material was also associated with requesting the use of condoms during sexual activity, talking about HIV/AIDS with partners, friends and family, and with supportive and help-seeking behaviours.

A Kenyan study used content analysis to explore whether an entertainment-education drama called ‘Shuga’ conveyed its intended messages.^120^ Shuga was intended to promote safe sex by encouraging condom use and monogamy and to destigmatise HIV/AIDS. Content analysts coded 138 min from six episodes of Shuga and found that the series conveyed strong and consistent destigmatising and condom-promoting messages. However, the analysts’ findings suggest that messaging promoting monogamy as a component of safe sex practice was neither strong nor consistent, with few direct statements of this message, a majority of characters engaged in multiple concurrent partnerships, and a lack of a role model couple engaged in monogamous sexual partnership.

### Visual art (other), digital art, comics

Visual arts were present in 11 of the identified references.^90 122–131^ One paper described a three-phase piece of work which used sculpture as an HIV/AIDS awareness-raising and prevention strategy in Uganda.^128^ In each phase, a professional sculpture artist created pieces of sculpture that sought to reflect on and challenge taboos surrounding the discussion of HIV/AIDS, sex and death. The study culminated in a series of soap-based sculptures, a medium chosen for its portability, allowing the sculptures to be moved, exhibited and handled in communities across Uganda. This sculpture-based approach was evaluated through interviews and focus groups with members of the community in which the sculptures were exhibited. The qualitative data gathered suggested that the sculptured imagery was seen to be readily intelligible, evoking lots of interpretations that ‘fit’ with the health-promoting agenda: the vulnerability of the body, which diminishes with use, like soap; the linking of genitalia, sex and infection; embedding objects in genitalia evoke repulsion at the invasion/infection.

We found five papers which used a form of digital art.^117 129 131–133^ With the spread of video-capable mobile phones, one group of researchers used this platform to explore the use of digital animations as a medium for providing health education and prevention messages relating to insect control, and cholera and malaria prevention in Benin.^132^ A 3D animated video, presented in local language, was created for each target topic and shown twice to participants using mobile phones, who then completed a short survey. Of the 83 participants, 100% ‘liked’ the video, and all but one rated the ‘message’ as ‘clear’. The authors conclude that this approach to health promotion is feasible and, as mobile technologies continue to spread, could also provide an approach that is scalable.

Comics were deployed in four of the studies we identified.^109 134–136^ One study of Swahili language HIV/AIDS prevention comics uses a case-study approach to contrast the content of a UNICEF-funded comic to that of a locally funded and produced comic.^134^ The study’s author’s discourse analysis claims that the UNICEF-funded comic relies on a simplistic ‘goodies’ versus ‘baddies’ narrative structure, while the locally produced comic uses ambiguity to enable readers to identify with the characters. The author concludes that the UNICEF comic underestimates both the medium of the comic and the young East-African readers it is aimed at, and that until its shortcoming are addressed, it is not a suitable approach to HIV/AIDS prevention.

### Film, photography, photovoice

Six studies explored the use of film to promote health,^103 137–141^ one of which used a multiform approach. In Tanzania, a research team used participatory research methods to create video content to reflect on and challenge the reproductive health issues faced by adolescents.^140^ The study team worked with gender-balanced groups of school-going children aged 15–19 years, to identify and prioritise reasons why teenagers become pregnant, then writing scripts that worked with the prioritised reasons to create screenplays. Participants then worked with the research team to shoot and edit footage of their screenplays to produce films which were shown to policy-makers, broadcast on local television and shared through YouTube. Reflecting on their work, the author suggested that participatory video approaches can be a useful means of engaging young people in health issues, providing voice to their experience and empowering them. However, the author also noted that the researchers experienced considerable challenges relating to the power dynamics between the research team and the participants. Specifically, the participants argued that they should be paid a salary, as they saw their involvement in the project as work. This issue was resolved by providing a leisure event at the end of the project.

Photography and photovoice approaches to health promotion were used in eight papers.^135 142–148^ Researchers in Mozambique used photographs in combination with vignettes to elicit social norms relating to multiple concurrent sexual partnerships, with the aim of contributing to HIV/AIDS prevention efforts.^143^ The authors report that by using projective techniques, they were able to elicit rich narratives relating to sexual norms, HIV/AIDS transmission and risk perception, and that this method can be particularly useful during the development phases of interventions, allowing researchers to access local constructs and terminologies without imposing external assumptions.

In South Africa, Mitchell *et al* used a photovoice and participatory research methodology to facilitate community health workers and teachers to represent and respond to HIV/AIDS in their communities.^144^ Over the course of four sessions, participants formed groups, were taught to use cameras, encouraged to take pictures of the challenges and potential solutions for addressing HIV/AIDS-related issues, and gathered together to view and discuss the pictures that had been taken. The pictures and discussions provided insight into local experiences of HIV/AIDS but were also a platform for community organisation and mobilisation, with participants using their newly formed social networks as a platform to instigate stigma-reduction initiatives and education programmes.

### Storytelling

Of the nine papers that worked with storytelling,^60 127 129 133 149–153^ nine (55.5%) approached the method independently, and four (44.4%) as part of a multiform approach. Two studies describe a storytelling competition, in which young people were invited to submit HIV/AIDS-related narratives designed for transformation into short films.^150 153^ The competitions reached 63 327 young people and received 22 894 screenplays, and the researchers suggested that the narratives conveyed that HIV/AIDS stigma was common, the virus is associated with sexual promiscuity, that antiretrovirals (ARVs) are viewed with ambivalence, and that social support networks for those living with the virus are limited. A further study presented a critical analysis of the Zimbabwean HIV/AIDS intervention strategy for schools, revealing that non-fictional texts dominated and argued that fictional texts should be included to help pupils grapple with the social and emotional dimensions of the epidemic.^149^

In a workplace setting, and using participatory workshops, South African researchers collaborated with employees of a large mining company to tackle HIV/AIDS myths using stories developed with peer educators.^151^ A total of 80 variations of myth were identified in the initial workshops but in later workshops, the researchers found that some of peer educators struggled to create appropriate stories to counter these myths. The researchers concluded that while not all peer educators found it easy to create alternative HIV/AIDS narratives, those who did created richer stories, more suitable to their context, than the storytelling experts could on their own.

A university in South Africa was also the site for testing storytelling-based HIV/AIDS awareness and prevention messaging.^152^ Using a small sample of 21 students, researchers tested the effect of a prerecorded ‘storytelling stimulus’ on self-reported intentions relating to HIV/AIDs-related risks. The study found statistically significant relationships between participants’ levels of interest in the story and their ability to recall its content, and between their levels of absorption in the story and their intention to carry out at least one positive behaviour.

Multiform approaches that used storytelling tended to include digital or visual arts. In one South African study, which was part of a narrative therapy initiative for women living with HIV/AIDS, participants combined storytelling with the creation of body maps, through which they narrated experiences and histories relating to their condition.^127^ Two school-based studies, one in Tanzania^129^ and one in South Africa,^133^ used a combination of storytelling, visual arts and digital arts to support students to produce stories layered with images, drawings and spoken words. These stories were used to facilitate HIV/AIDS education and social-behavioural change, including challenging stigma.

### Folk media, craft

Folk media^56 65 81 108^ and craft^75 123 124 154^ were each referred to in four papers. All but one^154^ of these papers discussed multiform work. Folk media was generally used as an umbrella term to refer to art and expressive forms derived from the specific community in which the work was being conducted. One study in Malawi highlighted the complexities of this approach, demonstrating that what researchers may assume to be a ‘local’ form of expression can, if not carefully negotiated with target communities, in fact turn out to be external and alienating.^81^ The paper notes that folk dances are particularly problematic in this regard, as the meanings and practices that surround specific dances can vary greatly, even within cultural groupings that are assumed to have a degree of homogeneity.

In South Africa, one study presents a project that worked with rural women to produce beaded craft works and telephone wire *imbenge* (pot covers and baskets) carrying HIV/AIDS messaging conceived of by participants.^123^ Alongside work on mural and billboard campaigns, the paper presents bead-based craft as a means to challenge social taboos. The women who participated in the craft project produced objects, such as tableaux, depicting promiscuity or that emphasised unprotected sex, which challenged traditional Zulu taboos relating to explicit communication about sexuality and love. The author defends investment in arts as a means of preventing and raising awareness of HIV/AIDS, suggesting that they are more acceptable to local populations than traditional education programmes, adding that such investment can also beautify the environment, provide employment and encourage activism.

### Circus, comedy, puppetry

Single papers each described the use of circus arts,^60^ comedy^61^ and puppetry^40^ in combination with other art forms. South Africa’s ‘Clowns Without Borders’ organisation used circus arts in combination with theatre, storytelling and song with the aim of improving the well-being of children orphaned by HIV/AIDs in South Africa, Lesotho and Swaziland.^60^ This multiform intervention was initially deployed as a brief intervention, before the organisers moved to a residency-based programme in collaboration with local NGOs to improve sustainability. The author of the study reports that while monitoring and evaluation work is underway, no formal evaluation is being conducted.

Comedy was used as part of another multiform intervention in Ghana.^61^ Following the Ghanaian performance tradition of ‘concert party’, the researchers worked with a combination of popular Ghanaian artists and people living with HIV to present an HIV/AIDS education event. Comedy acts were used to ‘break the ice’ with audience, by openly joking about relationships, sex and condoms; topics which, in the Ghanaian communities that participated in the research, are not usually spoken of publicly.

During the early 1990s, researchers in South Africa used a combination of puppetry and street theatre to deliver an HIV/AIDS education programme in South Africa.^40^ Larger than life, puppets told the story of how a single person with HIV transmits the virus to multiple others before dying. The study aimed to inculcate a realistic fear of HIV/AIDS, communicate that changes in behaviour can be protective, and encourage audience members to be supportive of those who contracted the virus. The researchers used pre- and post- performance questionnaires to assess the programme and detected statistically significant short-term improvements in knowledge and intention.

## Discussion

Our review has identified the wide range of arts-based approaches that have been used within health promotion research in SSA. Theatre-based work was the most prominent among these approaches, but music and song, TV and radio, visual arts, storytelling and film-based work have all been explored, among others. The majority of these approaches have been mobilised as part of public health responses to the HIV/AIDS epidemic, but Ebola and malaria have also received some sustained attention. Other health conditions and issues have been explored with arts-based approaches, but in a more sporadic and isolated manner. The geographical spread of the research work is wide, with 30 of the 48 countries in the SSA region represented. However, as found in reviews of literature on other health-related topics in the region, South African-based research dominates.^155^

A central feature of art-based methods for health is their capacity for use during research enquiries.^14 156^ Reflecting debates that began as far back as ancient Greece,^157^ arts are positioned by some researchers as an alternative mode of knowledge generation that can reveal dimensions of human experience that are difficult to represent with words.^158^ Papers included in our review suggest that this approach has been used with some success in the SSA region. Storytelling through the drawing of body maps, as presented by Horne, enabled Xhosa-speaking women living with HIV to envisage their bodies and represent histories that had often remained untold.^127^ Similarly, Molesante *et al*’s school-based study in South Africa demonstrates how photovoice techniques can elicit and reveal experiences of HIV/AIDS-related stigma.^145^ In both of these papers, however, art as enquiry is coupled with explorations of the effect the enquiry had or could have for participants and their communities. In doing so, the authors acknowledge the long-held position that art cannot be contained within a representational framework alone and can set change and transformation in motion. In future studies in the SSA region, there is an opportunity to deepen understandings of how arts-based enquiries affect those who participate and the actions such enquiries might facilitate, intentional or otherwise.

One of the significant achievements of arts-based approaches in SSA has been to reach and engage large numbers of people in health-focused reflection and action. The entertainment-education approaches applied in TV and radio have proven to be particularly capable of reaching large numbers with HIV/AIDS-related materials.^111 115^ Theatre-based work has also been shown to attract large numbers of people, both as audiences and as participants in organisations and initiatives, which have been characterised as a social movement.^57^ During Ebola outbreaks, popular^25^ and traditional^101^ music has also been used to reach large audiences. With the SSA region continuing to face a double burden of infectious diseases and non-communicable diseases (NCDs),^159^ these findings suggest that TV, radio, theatre and music are important tools for reaching populations and engaging them to reflect on health-related practices.

As well as offering novel modes of enquiry and reaching large populations, some arts-based approaches have been able to succeed in engaging their participants in changes which are beneficial for health. Street theatre performances have been strongly associated with large increases in self-referrals for HIV/AIDS screening^15^ and both TV^111^ and radio^115^ edutainment approaches show positive associations with a range of self-reported HIV/AIDS-related measures. While some studies have suggested that identifying with characters is a key pathway through which change might be inculcated in story-based art forms,^152^ few papers in the review theorise and study how change takes place in response to arts-based interventions. Panter-Brick and colleagues’ song and poster-based malaria prevention work is an exception and highlights how an approach grounded in indigenous cultural forms and musical traditions can support significant improvements in bed net repairs.^90^ The authors argue that by working with their participants’ musical and visual traditions the approach was not only culturally ‘acceptable’ or ‘competent’ but also culturally ‘compelling.’ Future research in the SSA region has the opportunity to build and test theories of arts-mediated health-related change.

The content of arts-based approaches to health promotion has not always been ‘compelling’ in the manner described by Panter-Brick and colleagues. Indeed, some studies report that addressing sex and related practices through theatre can be met with negative responses relating to taboos.^59^ Another challenge relating to content is well-illustrated in a study of the edutainment soap *Shuga*, which suggested that the show reinforced rather than challenged the practice of taking multiple concurrent sexual partners.^120^ Finally, as highlighted by Abdulla, arts-based approaches have to pay careful attention to the specificity of cultural forms, such as dance, used during health-promoting activities.^81^ What is assumed to be ‘of’ a community may in fact be alien, and therefore be treated as a spectacle and not met with participation. Researchers considering how to work with arts-based approaches to health promotion in SSA should reflect on these content-related challenges and anticipate how to address them.

While many studies present positive accounts of arts-based approaches that have enabled participation in health agendas, some sound notes of caution and point to issues of power. Papers such as Beck’s study of comic-based approaches to HIV/AIDS prevention demonstrate that, while acting with good intentions, international agencies have imposed narratives which are unsophisticated and unappealing and lack the subtly and realism of local comic forms.^134^ Such top-down approaches have also been taken via popular music and TV, and risk presenting narrative which fail to resonate with audiences, or worse, inculcate resistance to engagement with information and issues of great importance for health. When arts-based approaches do work with local communities to produce minimally directed content, issues of inequalities between researchers and participants can arise. Specifically, participants can feel that the modest allowances they receive from researchers inadequately compensate them for the time they invest in producing art works. Such power struggles can be heightened and intensified when the researchers working in SSA territories are white Europeans, Americans or other from other counties associated with wealth.^140^ Drawing on the work of Ellsworth, Kennedy Chinyowa encapsulates issues of power in arts-based approaches to health promotion by exploring participation as a form of ‘repressive myth’. He argues that when artists and researchers fail to put participants ‘at the centre of the intervention process’, they place their own interests first, silencing instead of empowering them.^80^ Such perspectives serve as a stark warning to researchers engaging in arts-based health promotion in contexts riven with memories of biomedicine’s impositions.^160^

A final challenge identified in the literature relates to how arts-based approaches to health promotion have been evaluated. This challenge is by no means peculiar to the SSA region and has been highlighted by researchers from the global north, and very few studies employ research designs capable of producing the types of evidence conventionally accepted by medical and policy professionals.^23 161^ While our review has shown that statistical assessment of arts-based health promotion has been conducted in SSA territories, these assessments are usually limited to self-report before-and-after designs.^45^ Evaluation via qualitative approaches to data collection often renders extremely positive accounts of arts-based initiatives, but these accounts sometimes appear overstated, particularly when they fail to consider negative and unintended consequences. Innovative approaches to evaluating future arts-based approaches to health promotion in SSA are clearly needed and while experimental and statistical assessments should be considered, researchers should think critically about the tools they use to assess the impact of methods that champion aspects of experience and life often considered to be intangible, yet vital.^162^

### Strengths and limitations

The review we have presented here is limited by four factors. First, the searches we conducted were restricted to English language outputs. As a consequence, our findings predominantly relate to the Anglophone territories of SSA. Second, we chose to confine our searches to articles published in peer-reviewed journals. This choice facilitated international collaboration, allowing all retrieved articles to be shared electronically across the group. In this review, we have engaged with some of the many book-based contributions to the field, but we acknowledge that we have not done justice to the breadth of these contributions and have completely omitted grey literature. Third, the narrative approach we have taken to reporting our findings limits the systematicity of our reporting but enabled a multidisciplinary team to participate equitably in the review process.

Fourth, while the choice to locate our review within the WHO-defined tradition of health promotion is defensible in the context of our research question, a consequence is that we did not capture studies with a primary focus on art-based research, therapy or knowledge translation. Future work should explore how these other traditions have been approached in the SSA region and how practitioners and researchers in these traditions might enter into productive dialogue with African researchers. Finally, following the scholarship of authors such as Okagbue^107^ and Kerr,^19^ we note that the categorisation of art forms we have employed is derived from the Western cannon, and does not reflect African conceptualisations of performance, which tend not to make as many distinctions between forms.

## Conclusions

It is clear from this review that arts-based approaches to health promotion have generated a rich academic literature relating to a wide range of SSA territories. The overwhelming majority of studies have focused on HIV/AIDS, pursuing prevention, testing and destigmatisation agendas. The learning contained in these studies has significant potential to inform public health responses to the growing burden of NCDs in SSA, as well as other health-related problems. Specifically, the literature we have identified suggests that there is potential for future research efforts to use arts-based approaches to reach large populations, drive enquiries into health-related problems and facilitate reflection on and change in health-related issues. While often championed by participatory researchers, arts-based approaches are not immune to the problem of imposition, can transmit content and messages that are inappropriate or offend, and pose challenges for evaluators. With care and focus on the communities with which engagement is sought, arts-based approaches to health promotion clearly have a useful role to play in improving public health in relation to both communicable disease and NCD in SSA and present opportunities for researchers to develop and evaluate innovative theory-informed work.
