# Supplementary material for: Arts-based approaches to promoting health in sub-Saharan Africa: a scoping review
Source: BMJ Glob Health. 2020 May 21;5(5):e001987. doi: 10.1136/bmjgh-2019-001987 (PMC7247409; doi:10.1136/bmjgh-2019-001987)
Supplement: Supplementary data [file bmjgh-2019-001987supp001.pdf]

Supplementary Table 1:

Focus	Terms
Health promotion topic	“acquired immune deficiency syndrome” OR “AIDS” OR “asthma*” OR “breast feeding” OR “blood pressure” OR “bronchitis” OR “cancer” OR “cardiovascular disease” OR “child health” OR “cholera” OR “chronic kidney disease” OR “chronic lung disease” OR “COPD” OR “Diabet*” OR “HIV” OR “human immunodeficiency virus” OR “heart disease” OR “hypertension” OR “immune*” OR “infant health” OR “iodine” OR “leprosy” OR “malari*” OR “maternal health” OR “NCD” OR “non-communicable disease*” OR “nutrition” OR “polio” OR “pneumonia” OR “rehydration” OR “respiratory disease” OR “sex*” OR “schistosomiasis” OR “smallpox” OR “stroke” OR “syphilis” OR “TB” OR “tetanus” OR “trachoma” OR “tuberculosis” OR “vaccination” OR “vitamin” OR “zoono*”<br><br>AND
Arts and practices	“artisan” OR “carving” OR “ceramic” OR “collage” OR “comedy” OR “comic” OR “cultur*” OR “danc*” OR “digital” OR “drama” OR “draw*” OR “film” OR “folk media” OR “game*” OR “literature” OR “music” OR “paint*” OR “photog*” OR “play” OR “poem” OR “poet*” OR “poster*” OR “pottery” OR “sing” OR “song” OR “story” OR “theatre” OR “writing”<br><br>AND
Location	“Abyssinia “ OR “Africa” OR “Sub-Saharan Africa” OR “Angola” OR “Bechuanaland” OR “Benin” OR “Botswana” OR “British Central Africa” OR “Burkina Faso” OR “Burundi” OR “Cabo Verde” OR “Cameroon” OR “Cape Verde” OR “Central African Republic” OR “Chad” OR “Comoros” OR “Costa da Pimentia (Pepper Coast)” OR “Dahomey” OR “Democratic Republic of the Congo” OR “Djibouti” OR “East Africa Protectorate” OR “Equatorial Guinea” OR “Eritrea” OR “Ethiopia” OR “French Equatorial Africa” OR “French Guinea” OR “French Territory of the Afars and the Isas” OR “French West Africa” OR “Gabon” OR “Gambia” OR “Ghana” OR “German East Africa” OR “Gold Coast” OR “Guinea” OR “Guinea-Bissau” OR “Ivory Coast” OR “Kenya” OR “Lesotho” OR “Liberia” OR “Madagascar” OR “Malawi” OR “Mali” OR “Mauritania” OR “Mauritius” OR “Mozambique” OR “Namibia” OR “Niger” OR “Nigeria” OR “Northern Rhodesia” OR “Nyasaland” OR “Portuguese Guinea” OR “Protectorate of Togoland” OR “Republic of the Congo” OR “Rhodesia” OR “Rio Nuni and Fernando Poo” OR “Ruanda-Urundi” OR “Rwanda” OR “Senegal” OR “Seychelles” OR “Sierra Leone” OR “Somalia” OR “South Africa” OR “South Sudan” OR “South West Africa” OR “Southern Rhodesia” OR “Sudan” OR “Sudanese Republic” OR “Swaziland” or “São Tomé and Príncipe” OR “Tanganyika & Zanzibar” OR “Tanzania” OR “Territory of Basutoland” OR “The Kenya Colony” OR “Togo” OR “Uganda” OR “United Republic of Tanganyika and Zanzibar” OR “Union of South Africa” OR “Upper Volta” OR “Zaire” OR “Zambia” OR “Zimbabwe” OR “Zimbabwe Rhodesia”

Supplementary Table 2: Number of records identified by database

DATABASE NAME	NUMBER OF RECORDS RETURNED
Anthropology plus	629
ARTbibliographies Modern (ABM)	955
Arts and Humanities Database	8,869
CINAHL	3,080
Education Abstracts	629
International Bibliography of Social Sciences	9,848
MEDLINE	22,491
MLA International Bibliography	626
Music Periodicals Database	2,831
PsychINFO	7,288
SocINDEX	2,548
**Total**	**59,794**

Supplementary Table 3: List of records included in the review

Publication Year	First Author	Location	Health Issue	Art form
2016	Abdulla[70]	Malawi	HIV/AIDS	Dance, Folk Media
2009	Allen[114]	South Africa	HIV/AIDS	Visual Arts
2009	Bastien[83]	Tanzania	HIV/AIDS	Music and Song
2006	Beck[122]	Tanzania, Kenya, an other SSA countries	HIV/AIDS	Comics
2015	Bekalu[107]	Ethiopia	HIV/AIDS	TV/Radio
2015	Bekalu[88]	Ethiopia	HIV/AIDS	Music and Song
2013	Bello-Bravo[120]	Benin	Cholera and Malaria	Digital Art
2003	Biella[125]	Southern Africa region	HIV/AIDS	Film
2013	Bilbrough[52]	South Africa	HIV/AIDS	Theatre
2014	Black[86]	South Africa	HIV/AIDS	Music and Song
2015	Black[89]	South Africa	HIV/AIDS	Music and Song
2011	Boneh[50]	Ghana	HIV/AIDS	Theatre, Comedy, Music and Song
2013	Boneh[53]	Malawi and Ghana	HIV/AIDS	Theatre
2016	Booker[108]	Kenya	HIV/AIDS	TV/Radio
1991	Bosompra[27]	Ghana	HIV/AIDS	Theatre, Music and Songs
2006	Chinyowa[15]	Zimbabwe	HIV/AIDS	Theatre
2007	Chinyowa[43]	Zimbabwe	HIV/AIDS	Theatre
2009	Chinyowa[45]	South Africa and Zimbabwe	HIV/AIDS	Theatre and Folk Media
2009	Chinyowa[46]	South Africa	HIV/AIDS	Theatre
2013	Chinyowa[54]	N/A	HIV/AIDS	Theatre and Folk Media
2015	Chinyowa[69]	South Africa	HIV/AIDS	Theatre
2013	Chipatiso[55]	South Africa	HIV/AIDS	Theatre
2013	Chivandikwa[56]	Zimbabwe	HIV/AIDS	Theatre
2006	Dalrymple[39]	South Africa	HIV/AIDS	Theatre
1993	Dalrymple[29]	South Africa	HIV/AIDS	Theatre
2011	Dickinson[139]	South Africa	HIV/AIDS	Storytelling
2005	Dos Santos[78]	South Africa	HIV/AIDS	Music and Song
2015	Doubt[119]	South Africa	HIV/AIDS	Digital Art and Visual Art
2016	Duke[71]	Nigeria	Childhood cataracts	Theatre
2012	Durden[51]	South Africa	HIV/AIDS	Theatre

2013	Duveskog[117]	Tanzania	HIV/AIDS	Visual Arts, Digital Arts, Storytelling
2003	Englehart[126]	South Africa	HIV/AIDS	Film
2005	Farr[98]	Ethiopia	HIV/AIDS	TV/Radio
2017	Fayoyin[91]	South Africa	Ebola and Family Planning	Music and Song
2014	Flax[66]	Nigeria	Breastfeeding	Music and Song, Theatre
2007	Fouché[80]	South Africa	HIV/AIDS	Music and Song
2014	Fournier[136]	Uganda	HIV/AIDS	Photovoice
2006	Francis[112]	South Africa	HIV/AIDS	Visual Arts, Craft
2010	Francis[47]	South Africa	HIV/AIDS	Theatre
1996	Frank[30]	Uganda	HIV/AIDS	Theatre
2016	Frishkopf[72]	Ghana	Cholera and Malaria	Music and Song, Theatre, Dance
2017	Frishkopf[92]	Liberia	Water, sanitation, and hygiene	Music and Song, TV/Radio, Film
2005	Goldstein[99]	South Africa	HIV/AIDS	TV/Radio
2003	Gunner[77]	South Africa	HIV/AIDS	Music and Song
2000	Harvey[33]	South Africa	HIV/AIDS	Theatre
2016	Holman[131]	Mozambique	HIV/AIDS	Photography
2011	Horne[115]	South Africa	HIV/AIDS	Storytelling, Visual Art
2008	Isabirye[81]	Uganda	HIV/AIDS	Music and Song
2008	Jacobs[134]	South Africa	HIV/AIDS	Photovoice
2013	Jaganath[67]	Malawi	HIV/AIDS	Theatre
2010	Johansson[48]	Tanzania	HIV/AIDS	Theatre
2008	Kamo[44]	Tanzania	HIV/AIDS	Theatre
2013	Kerr[57]	Botswana, Malawi	HIV/AIDS	Theatre
2002	Kincaid[35]	N/A	HIV/AIDS	Theatre
1997	Kottler[32]	South Africa	GBV	Theatre
2007	Lovell[100]	Botswana	HIV/AIDS	TV/Radio
2002	Mabala[36]	Tanzania	HIV/AIDS	Theatre
2015	Makalela[140]	South Africa	HIV/AIDS	Storytelling
2013	Mambwe[85]	Zambia	HIV/AIDS	Music and Song
2013	Mangeni[58]	Uganda	HIV/AIDS	Theatre
2004	Mapara[110]	Botswana	HIV/AIDS	Visual Arts
2004	Marschall[111]	South Africa	HIV/AIDS	Visual Arts, Craft

2006	Mbizvo[40]	Cameroon, Namibia, Togo, Zambia, Malawi, Zimbabwe and Nigeria	HIV/AIDS, childhood immunisations, measles, malaria	Theatre
2004	Mbuyazi[37]	South Africa	HIV/AIDS	Theatre
2010	McClaren[49]	South Africa, Lesotho and Swaziland	HIV/AIDS	Circus Arts, Theatre, Music and Songs, Storytelling
2016	McConnell[90]	The Gambia	Ebola	Music and Song
2017	McConnell[93]	The Gambia	Ebola	Music and Song
2008	McNeill[82]	South Africa	HIV/AIDS	Music and Song
2006	Middelkoop[17]	South Africa	HIV/AIDS	Theatre
2005	Mitchell[132]	South Africa	HIV/AIDS	Photovoice
2015	Mnisi[121]	South Africa	HIV/AIDS	Digital Art, Storytelling
2013	Mokuku[59]	Lesotho	HIV/AIDS	Theatre
2007	Moletsane[133]	South Africa	HIV/AIDS	Photovoice, Theatre
2013	Mtukwa[60]	South Africa	HIV/AIDS	Theatre
2011	Mutonyi[124]	Uganda	HIV/AIDS	Comics
2010	Nabulime[116]	Uganda	HIV/AIDS	Visual Arts
2007	Ngoshi[137]	Zimbabwe	HIV/AIDS	Storytelling
2007	O'Leary[101]	Botswana	HIV/AIDS	TV/Radio
2001	Panford[96]	Ghana	HIV/AIDS	TV/Radio, Folk media
2005	Panter-Brick[79]	The Gambia	Malaria	Music and Song, Visual Art
20008	Pappas-DeLuca[103]	Botswana	HIV/AIDS	TV/Radio
2017	Parent[74]	South Africa	TB	Theatre
2013	Perlman[105]	South Africa	HIV/AIDS and GBV	TV/Radio, Digital Art
2013	Pfeiffer	Tanzania	Family Planning	Film
2008	Rimal[104]	Malawi	HIV/AIDS	TV/Radio
2004	Risi[97]	South Africa	Cervical cancer	Comics, TV/Radio
2017	Rivera[94]	Liberia	Ebola	Music and Song
2001	Roberts[142]	South Africa	HIV/AIDS	Craft
2013	Rugg[61]	Swaziland and South Africa.	HIV/AIDS	Theatre
2004	Rumsey[38]	Malawi	HIV/AIDS	Theatre, Music and Songs
2016	Ruthven[73]	South Africa	HIV/AIDS	Theatre
2017	Ruthven[75]	South Africa	HIV/AIDS	Theatre
2006	Samba[41]	Cameroon	HIV/AIDS	Theatre, music and songs, poetry
2013	Samba[62]	SSA	HIV/AIDS	Theatre

1996	Seguin[31]	Mali	Women's Health, HIV/AIDS	Theatre
1999	Sicherman[34]	Uganda	HIV/AIDS	Theatre and Dance
1991	Skinner[28]	South Africa	HIV/AIDS	Theatre and Puppetry
2011	Skovdal[130]	Kenya	HIV/AIDS	Photography
2007	Smith[102]	Ethiopia	HIV/AIDS	TV/Radio
2014	Smith[87]	South Africa	CVD	Music and Song
2017a	Stone[22]	Liberia	Ebola	Music and Song
2017b	Stone[95]	The Gambia, Guinea, Liberia, and Sierra Leone	Ebola	Music and Song
2006	Stuttaford[42]	South Africa	Stroke	Theatre
2013	Sutherland[63]	South Africa	HIV/AIDS	Theatre
2013	Taub[64]	South Africa	HIV/AIDS	Theatre, Crafts
2006	Toroyan[123]	South Africa	HIV/AIDS	Photography, Comics
2014	Ukwen[106]	Nigeria	HIV/AIDS	TV/Radio
2008	Umurungi[135]	Rwanda	HIV/AIDS	Photovoice
2013	Uwah[65]	South Africa	HIV/AIDS	Theatre
2014	Uwah[68]	South Africa	HIV/AIDS	Theatre
2011	Waite[127]	Uganda	HIV/AIDS	Film
2017	Warheit[76]	Kenya	HIV/AIDS	Theatre
2011	Wenje[84]	Kenya	HIV/AIDS	Music and Song
2006	White[113]	South Africa	HIV/AIDS	Visual Arts
2014	Willis[129]	Zimbabwe	HIV/AIDS	Film
2013	Wilson[152]	South Africa	HIV/AIDS	Poetry
2009	Winskell[138]	35 African countries (but 13 'core countries' - Benin, Burkina Faso, Cape Verde, Guinea-Bissau, Kenya, Madagascar, Mali, Mozambique, Namibia, Niger, Nigeria, Swaziland, Senegal, Tanzania, Togo and Zambia).	HIV/AIDS	Storytelling
2015	Winskell[141]	Senegal, Burkina Faso, Nigeria, Kenya, Namibia and Swaziland	HIV/AIDS	Storytelling
2013	Wood[118]	Tanzania	HIV/AIDS	Visual Arts
